# Effects of Experimental Infection with *Lagovirus europaeus* GI.1 and GI.2 on the Fecal Microbiota of Rabbits: New Insights into Virus–Host Microbiota Associations

**DOI:** 10.3390/ijms27208963

**Published:** 2026-10-09

**Authors:** Beata Hukowska-Szematowicz, Andrzej Fitzner, Szymon Mazgaj, Andrzej Dybus, Hanna Kulig, Felix Shih-Hsiang Hsiao, Danuta Kosik-Bogacka, Yi-Hung Li, Yu-Hsiang Yu

**Affiliations:** 1 Laboratory of Infectious Biology, Molecular Biology and Immunology, Institute of Biology, University of Szczecin, st. Wąska 13, 71-415 Szczecin, Poland; szymon.mazgaj@usz.edu.pl; 2 Molecular Biology and Biotechnology Center, University of Szczecin, st. Wąska 13, 71-415 Szczecin, Poland; 3 Department of Virology and Viral Animal Disease, National Veterinary Research Institute—State Research Institute, st. Wodna 7, 98-220 Zduńska Wola, Poland; andrzej.fitzner@piwzp.pl; 4 National Reference Laboratory for Rabbit Hemorrhagic Disease (RHD), st. Wodna 7, 98-220 Zduńska Wola, Poland; 5 Doctoral School, University of Szczecin, st. A. Mickiewicz 16, 70-384 Szczecin, Poland; 6 Department of Genetics, West Pomeranian University of Technology in Szczecin, st. Aleja Piastów 45, 70-311 Szczecin, Poland; andrzej.dybus@zut.edu.pl (A.D.); hanna.kulig@zut.edu.pl (H.K.); 7 Department of Biotechnology and Animal Science, National Ilan University, No. 1, Sec. 1, Shennong Rd., Yilan City 26047, Taiwan; shhsiao@niu.edu.tw (F.S.-H.H.); yhli@niu.edu.tw (Y.-H.L.); yuyh@niu.edu.tw (Y.-H.Y.); 8 Department of Biology, Parasitology and Pharmaceutical Botany, Pomeranian Medical University in Szczecin, Powstanców Wielkopolskich 72, 70-204 Szczecin, Poland; danuta.kosik.bogacka@pum.edu.pl

**Keywords:** *Lagovirus europaeus*, fecal microbiota, RHDV, RHDV2, rabbit hemorrhagic disease, RHD, 16S rRNA, rabbits

## Abstract

*Lagovirus europaeus* GI.1 (RHDV) and GI.2 (RHDV2), the causative agents of rabbit hemorrhagic disease (RHD), are highly virulent pathogens of the European rabbit; however, their impact on the host fecal microbiota remains poorly understood. Previous field studies have suggested an association between the fecal microbiota and RHDV2 epidemiology in wild rabbits, highlighting the need for studies under controlled experimental conditions. To address this knowledge gap, we investigated the effects of experimental infection with *L*. *europaeus* GI.1 and GI.2 on the fecal microbiota of rabbits. Eighteen rabbits were assigned to three groups: an uninfected control group and two groups experimentally infected with *L. europaeus* GI.1 or GI.2 (*n* = 6 per group). Fecal microbiota composition was characterized by high-throughput sequencing of the V3–V4 region of the 16S rRNA gene using the HiSeq 2500 platform (Illumina). Rabbits infected with either genotype developed clinical signs consistent with RHD. No significant differences in alpha and beta diversity were observed. However, selective taxonomic shifts were detected, particularly in the GI.2 group, in which the relative abundance of Firmicutes was significantly higher and that of Bacteroidota significantly lower than in the control group. The abundance of Muribaculaceae was lower in both infected groups, whereas *Sporosarcina* reached its highest abundance in the GI.2 group. Correlation analysis revealed associations between selected bacterial taxa and clinical parameters. Muribaculaceae abundance was positively correlated with survival time and negatively correlated with lethargy and diarrhea, whereas *Sporosarcina* showed the opposite pattern, with positive correlations with lethargy and diarrhea and a negative correlation with survival time. Our findings indicate that experimental infection with *L. europaeus* GI.1 and GI.2 was not associated with a global restructuring of the fecal microbiota but rather with selective changes in the abundance of specific bacterial taxa, some of which were associated with viral genotype and the clinical course of infection. To our knowledge, this is the first controlled experimental study to directly compare the effects of *L. europaeus* GI.1 and GI.2 infection on the rabbit fecal microbiota. This study extends previous field observations by providing experimental evidence and offers new insights into the associations between *L. europaeus* infection and the host fecal microbiota during RHD.

## 1. Introduction

The European rabbit (*Oryctolagus cuniculus*) is ecologically, economically, and scientifically important. In Mediterranean ecosystems, it acts as a keystone species, influencing vegetation structure and constituting an important component of trophic networks [1]. At the same time, the European rabbit is one of the best-characterized animal models used in biomedical, immunological, and infectious disease research [2,3]. Despite its wide distribution, both wild and domestic rabbit populations remain particularly vulnerable to infections caused by lagoviruses responsible for rabbit hemorrhagic disease (RHD), which has represented one of the major threats to this species for more than three decades [4,5,6].

*Lagovirus europaeus* (*L. europaeus*) belongs to the family *Caliciviridae* and the genus *Lagovirus*. According to the current classification, lagoviruses within the species *L. europaeus* are classified into genogroup GI, comprising genotypes GI.1–GI.4 [7]. Among these, GI.1 (RHDV) and GI.2 (RHDV2) are of major epidemiological importance and differ in their biological properties, host range, and disease course [5,6,8]. The liver is the primary target organ of infection, and extensive viral replication is associated with hepatocellular necrosis and apoptosis, metabolic dysfunction, and disseminated intravascular coagulation (DIC) [5,6,9,10]. Differences in the dynamics of infections caused by *L. europaeus* GI.1 and GI.2 suggest that disease progression is determined not only by viral properties but also by complex host response mechanisms [11].

In this context, attention has increasingly focused on the gastrointestinal microbiota as a potential contributor to host homeostasis and responses to infection. The gut microbiota plays an important role in maintaining metabolic and immune homeostasis and preserving intestinal barrier integrity [12]. This is particularly relevant in rabbits, whose gastrointestinal physiology is closely linked to fermentation, occurring predominantly in the cecum, and to cecotrophy. The microbiota contributes to the degradation of complex plant polysaccharides and the production of short-chain fatty acids (SCFAs) and other metabolites that can modulate host metabolic and immune functions [13]. The fecal microbiota of the European rabbit is dominated by bacteria from the phyla Firmicutes and Bacteroidota, with lower relative abundances of representatives of Verrucomicrobiota, Proteobacteria, Cyanobacteria, Actinobacteria, and Mycoplasmatota [12,13,14,15,16]. Abundantly represented bacterial families include Lachnospiraceae, Ruminococcaceae, and Muribaculaceae, which include bacteria involved in complex carbohydrate metabolism and intestinal fermentation [12,13,14]. However, the composition of the rabbit microbiota is not static and may be shaped by numerous factors, including age, diet, gastrointestinal tract section, housing conditions, and host health status [12,13,14,15,16,17].

Despite growing interest in virus–host microbiota association, the impact of *L. europaeus* infection on the fecal microbiota remains poorly understood. To date, the most relevant evidence comes from Rouco et al. [18], who conducted a field study of a wild European rabbit population during natural outbreaks of *L. europaeus* GI.2 (RHDV2). The authors found that successive phases of the RHDV2 epidemic were accompanied by changes in bacterial community structure and the relative abundance of selected taxa, mainly from the phyla Bacteroidota and Firmicutes. Their findings suggested associations between the fecal microbiota profile and the epidemiological status of RHDV2, as well as between gut microbiome composition and RHDV2 epidemic dynamics. However, because the study was observational, it was not possible to determine whether the observed microbiota changes influenced host susceptibility to infection or represented secondary consequences of disease progression and population-level changes. The authors therefore emphasized the need for experimental models to disentangle these relationships while minimizing the influence of environmental, genetic, and individual-level factors [18].

The present study directly addresses this knowledge gap. In contrast to the observational approach employed by Rouco et al. [18], we used a controlled experimental model of rabbit infection with the two major *L. europaeus* genotypes, GI.1 and GI.2. This approach enabled, for the first time, a comparative assessment of infection-associated changes in the rabbit fecal microbiota while limiting the influence of environmental and population-level confounding factors. Thus, our study extends previous field observations by moving the investigation of the relationship between *L. europaeus* infection and the microbiota from an epidemiological setting to an experimental model. This approach allows not only the characterization of microbiota changes associated with infection but also the identification of bacterial taxa potentially associated with the clinical course of RHD. The study therefore provides new insights into virus–host microbiota associations during RHD and establishes a basis for further investigation of the microbiota’s functional significance and the potential utility of selected bacterial taxa as microbiological indicators of the RHD course.

This study aimed to assess the effects of experimental infection of rabbits with two genotypes of *L. europaeus*—GI.1 (RHDV) and GI.2 (RHDV2)—on the composition and diversity of the fecal microbiota and to determine the associations between the abundance of selected bacterial taxa and the clinical course of infection. We hypothesized that experimental infection with *L. europaeus* GI.1 (RHDV) and GI.2 (RHDV2) selectively modulates the composition of the rabbit fecal microbiota, leading to virus genotype-dependent changes in the abundance of specific bacterial taxa that may be associated with the clinical course of infection. To address this hypothesis, we used high-throughput sequencing of the V3–V4 region of the 16S rRNA gene on the Illumina HiSeq 2500 platform to characterize the composition (alpha and beta diversity) and structure (abundance) of fecal bacterial communities in European rabbits experimentally infected with *L. europaeus* GI.1 (RHDV) and GI.2 (RHDV2). To our knowledge, this is the first experimental study to directly compare the effects of *L. europaeus* GI.1 and GI.2 infection on rabbits’ fecal microbiota.

## 2. Results

### 2.1. Clinical Signs and Post-Mortem Findings

Animals infected with *L. europaeus* GI.1 (RHDV) and GI.2 (RHDV2) exhibited clinical signs characteristic of RHD, including loss of appetite, diarrhea, lethargy, tachypnea, elevated body temperature (>41 °C), and neurological signs. Selected clinical parameters, including the occurrence of diarrhea, lethargy, and loss of appetite, were analyzed in relation to animal survival time. Post-mortem examination of infected rabbits revealed gross pathological lesions in the internal organs characteristic of RHD. Mortality reached 100% in both infected groups; however, marked differences in disease dynamics were observed between the two genotypes. In rabbits infected with *L. europaeus* GI.2 (RHDV2), post-infection survival time ranged from 24 to 32 hpi, indicating a more rapid disease course, with the acute form of RHD predominating. In the *L. europaeus* GI.1 (RHDV)-infected group, survival time ranged from 32 to 60 hpi, although the acute form of RHD also predominated in this group (Table 1).

### 2.2. The Results of Microbiota Analysis

#### Fecal Microbiota Composition in Rabbits Infected with *L. europaeus* GI.1 (RHDV) and GI.2 (RHDV2)

**Figure 1:**
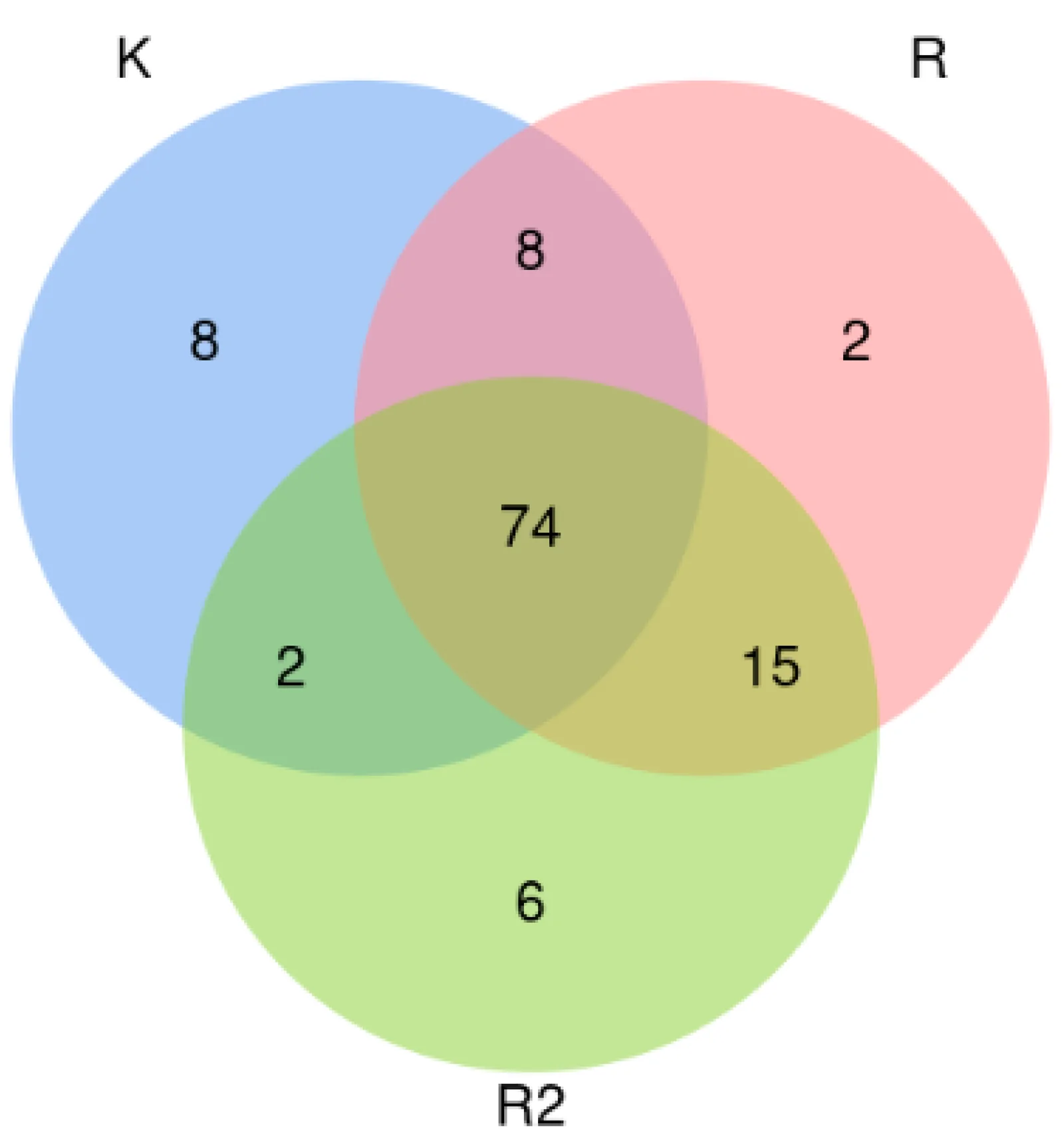
Venn diagram of the bacterial distribution in the fecal contents of rabbits. Each ellipse represents one group. The overlapping regions between the ellipses represent the bacteria shared among the following: healthy rabbits (K marking), rabbits infected with *L. europaeus* RHDV (R marking), and rabbits infected with *L. europaeus* RHDV2 (R2 marking) (*n* = 6). The value of each region represents the number of bacteria corresponding to the region.

Venn diagram analysis at the genus level showed that 74 bacterial taxa were shared among all three groups: the control group (K marking), the *L. europaeus* GI.1 (RHDV) (R marking), and the GI.2 (RHDV2) (R2 marking), constituting a shared core of the fecal microbiota (Figure 1). In addition, group-specific bacterial taxa were identified, with 2 unique taxa in group R, 6 in group R2, and 8 in group K. Analysis of taxa shared exclusively between pairs of groups revealed the greatest similarity between the two infected groups, R and R2, which shared 15 bacterial taxa. In comparison, groups K and R shared 8 taxa, whereas groups K and R2 shared only 2 taxa.

There was no significant difference in the alpha-diversity of the bacterial community between the groups (Table 2). An increasing trend of bacterial richness (Chao 1 estimator) was observed in the R2 group compared with the K group (*p* = 0.052). No significant differences were observed among the groups for the Fisher, Shannon, or Simpson indices (all *p* > 0.05).

**Figure 2:**
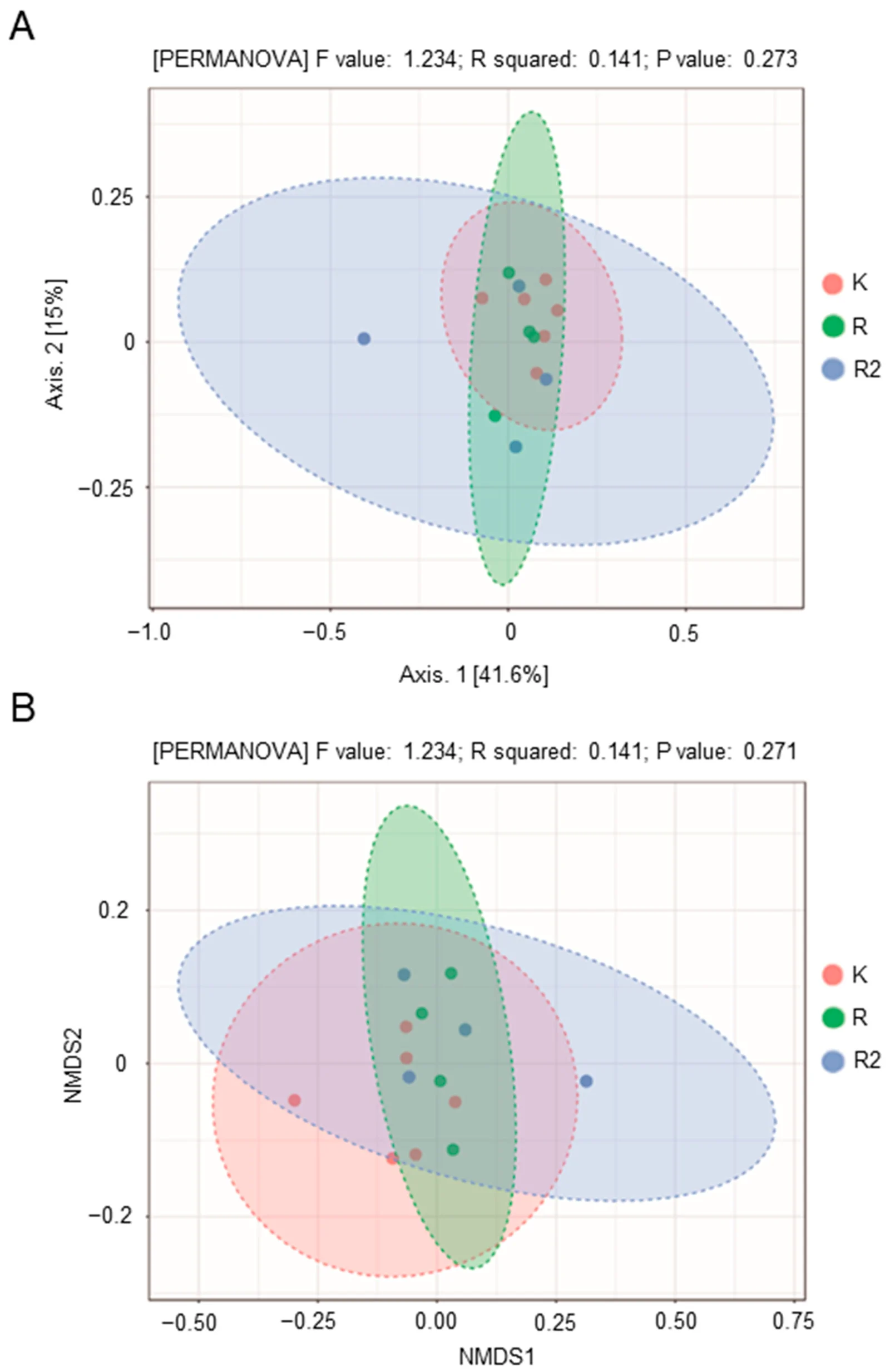
Effect of *L. europaeus* RHDV and RHDV2 infection on beta-diversity of microbiota in the fecal contents of rabbits. (**A**) Principal coordinate analysis of the fecal contents of healthy rabbits (K marking), rabbits infected with *L. europaeus* RHDV (R marking), and rabbits infected with *L. europaeus* RHDV2 (R2 marking) (*n* = 6). (**B**) Non-metric multidimensional scaling (NMDS) of the fecal contents of K, R, and R2 (*n* = 6).

Beta-diversity analysis of the bacterial communities using principal coordinates analysis (PCoA) and non-metric multidimensional scaling (NMDS) revealed no clear separation of fecal microbiota composition among the study groups (Figure 2A,B). The PCoA ordination showed substantial overlap among samples from the different groups, with no distinct clustering according to infection status (Figure 2A). PERMANOVA analysis confirmed that there were no significant differences in bacterial community structure among the groups. A similar pattern was observed in the NMDS analysis (Figure 2B), where samples from different groups partially overlapped and did not form clearly distinct clusters.

**Figure 3:**
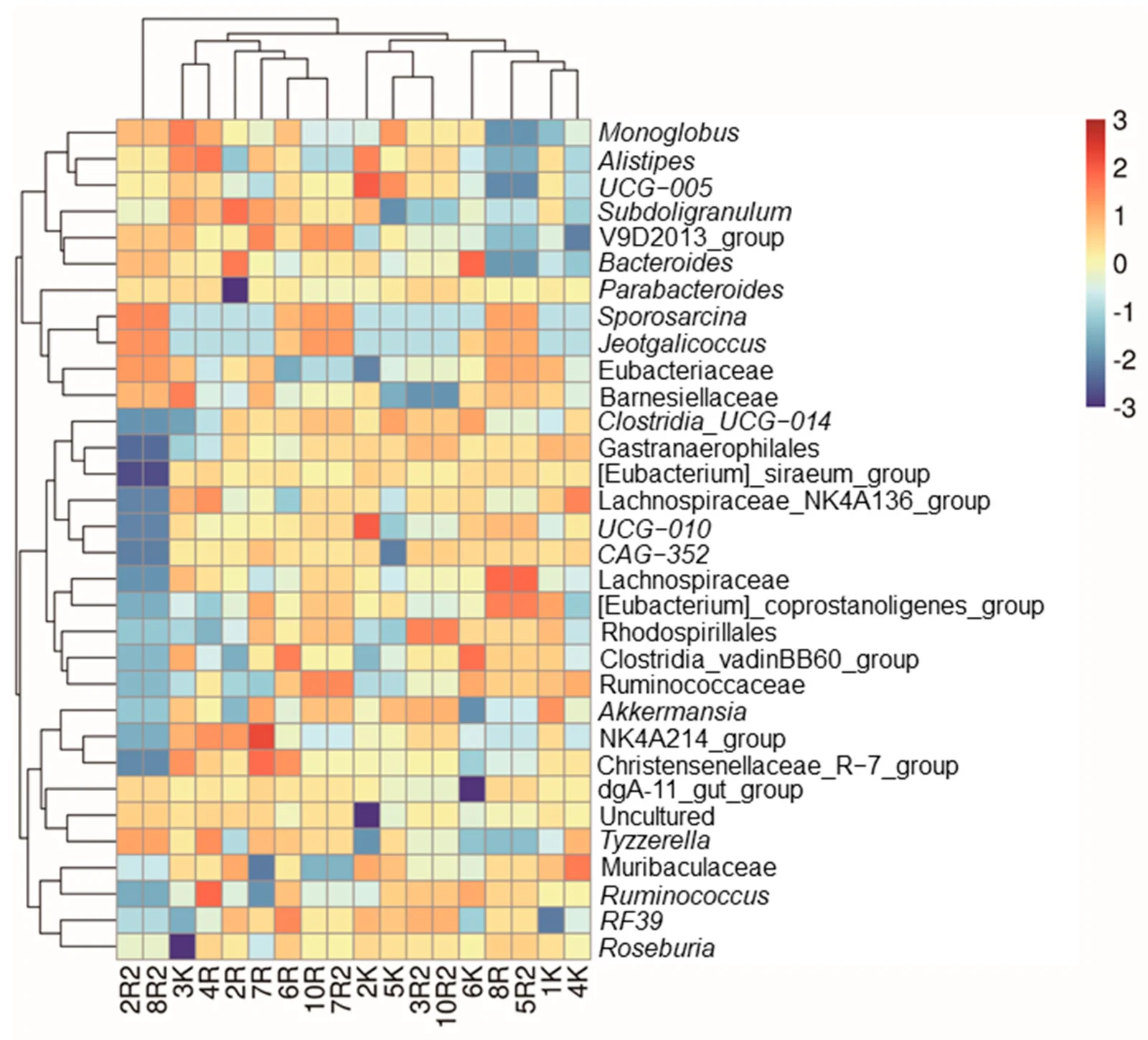
Heatmap of the abundance of dominant bacterial taxa in rabbit fecal samples. The distribution of 32 dominant bacterial taxa (Y-axis) across samples (X-axis) (*n* = 6) is shown. The values are normalized using the Z-score. Hierarchical clustering of samples and bacterial taxa is represented by dendrograms.

Based on the heatmap of the 32 most abundant genera (Figure 3), two genera (*Sporosarcina* and *Jeotgalicoccus*) were more abundant in the R2 group compared with the other groups. Similar bacterial clusters (NK4A214_group and Christensenellaceae_R-7_group) were abundant in the R group compared with the R2 group.

The effect of *L. europaeus* infection on the taxonomic composition of the rabbit fecal microbiota is presented in Table 3. Taxonomic analysis revealed that Firmicutes and Bacteroidota were the dominant bacterial phyla in all groups (K, R, R2). The abundance of Firmicutes was 64.0% in the control group (K), 71.4% in the GI.1-infected group (R), and 72.7% in the GI.2-infected group (R2). The abundance of Firmicutes was significantly higher in the R2 group than in the control group (*p* < 0.05). An opposite pattern was observed for Bacteroidota, whose abundance decreased from 26.3% in group K to 20.2% in group R and 17.8% in group R2. The difference between the R2 and K groups was statistically significant (*p* < 0.05). No significant differences among the groups were observed in the abundances of Cyanobacteria, Verrucomicrobiota, or Proteobacteria (*p* > 0.05).

Analysis of the selected bacterial taxa revealed no statistically significant differences in relative abundance among the experimental groups (Table 3). *Clostridia_UCG-014* and Muribaculaceae were among the dominant taxa. The abundance of Muribaculaceae was highest in the control group (17.3%) and lower in the infected groups, reaching 11.4% in group R and 9.0% in group R2. A trend toward a lower abundance of Muribaculaceae was observed in the R2 group compared with the R group (*p* = 0.063). *Sporosarcina* was not detected in the control group (0%), whereas its abundance was 0.9% in group R and 9.4% in group R2. A trend toward a higher abundance of *Sporosarcina* was observed in the R2 group compared with the K group (*p* = 0.050). No statistically significant differences among the groups were observed for the remaining analyzed bacterial taxa, including *Ruminococcus*, Clostridia_vadinBB60_group, Lachnospiraceae, Eubacteriaceae, V9D2013_group, Gastranaerophilales, NK4A214_group, *Bacteroides*, *Akkermansia*, *Alistipes*, Lachnospiraceae_NK4A136_group, and *UCG-010*.

**Figure 4:**
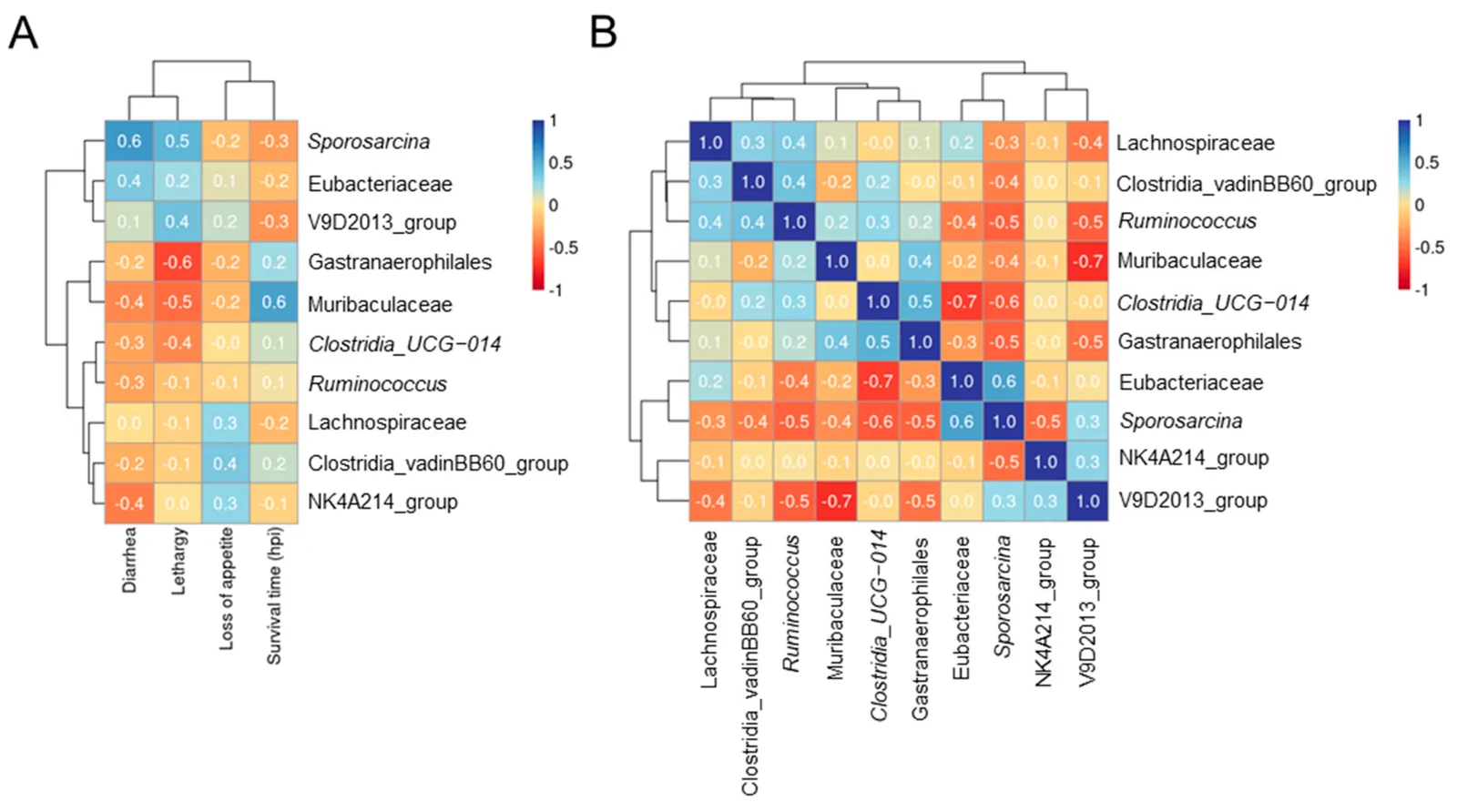
Correlation analysis of fecal microbiota of rabbits. (**A**) Correlation between the clinical signs of rabbits and dominant bacterial taxa in the fecal contents. (**B**) Correlations among dominant bacterial taxa in the fecal contents. Positive correlations are displayed in blue; negative correlations are shown in red. The values from +1 to −1 indicate the strength of the association.

Correlation analysis revealed associations between the relative abundance of selected bacterial taxa and the clinical parameters of infection (Figure 4A). The average abundance of the Muribaculaceae was positively correlated with the survival time of rabbits (Figure 4A). The average abundance of *Sporosarcina* and V9D2013_group was negatively correlated with the survival time of rabbits (Figure 4A). The average abundance of the bacterial taxa Lachnospiraceae, Clostridia_vadinBB60_group, and NK4A214_group was positively correlated with the loss of appetite in rabbits (Figure 4A). The average abundance of the taxa Eubacteriaceae, *Sporosarcina*, and V9D2013_group was positively correlated with lethargy (Figure 4A). The average abundance of the taxa Muribaculaceae, Gastranaerophilales, and *Clostridia_UCG-014* was negatively correlated with lethargy (Figure 4A). The average abundance of the Eubacteriaceae and *Sporosarcina* was positively correlated with diarrhea (Figure 4A). The average abundance of the taxa Muribaculaceae, *Clostridia_UCG-014*, *Ruminococcus*, and NK4A214_group was negatively correlated with diarrhea (Figure 4A).

Analysis of the relationships among the dominant bacterial taxa (Figure 4B) revealed both positive and negative correlations. The average abundance of the Muribaculaceae was negatively correlated with the abundance of the genera *Sporosarcina* and V9D2013_group (Figure 4B). The average abundance of the taxa Gastranaerophilales was positively correlated with the abundance of the *Clostridia_UCG-014* and the Muribaculaceae (Figure 4B). The average abundance of *Sporosarcina* was negatively correlated with the abundance of NK4A214_group (Figure 4B). The results indicate a complex network of positive and negative associations among the dominant components of the fecal microbiota.

## 3. Discussion

In this study, for the first time in an experimental model, the effect of infection with *L. europaeus*, two main genotypes—GI.1 (RHDV) and GI.2 (RHDV2)—on the fecal microbiota of rabbits was compared. The results indicate that infection did not cause a global restructuring of the bacterial community but was associated with selective changes in the abundance of specific bacterial taxa. There were no significant differences in alpha diversity and no clear group separation in the beta diversity analysis. The lack of clear separation between the experimental groups in the PCoA and NMDS analyses may be considered in the context of the clinical course of RHD, which could have influenced the time at which infection-associated changes became detectable in the fecal microbiota. Consequently, a significant number of bacterial taxa shared by the studied groups may partially reflect the dynamics of microbiota changes accompanying the course of RHD. The presence of 74 bacterial taxa shared among the control (K), GI.1 (R), and GI.2 (R2) groups indicates substantial overlap in fecal bacterial composition. This observation may also be considered in the context of the “core gut microbiota” concept described by Combes et al. [12], according to which certain components of the bacterial community are consistently present in the rabbit gastrointestinal tract and may constitute a functional core associated with host physiology and health. The larger number of taxa shared exclusively between the R and R2 groups may suggest some degree of similarity in the fecal microbiota response to infection. Consistent with the beta-diversity analyses, the heat map also did not reveal clear clustering of samples according to infection status. Despite the relative stability of the overall bacterial community structure, selective changes were observed at the taxonomic level, mainly affecting the dominant phyla Firmicutes and Bacteroidota and selected lower taxa. This effect was particularly visible in the GI.2-infected group, in which the relative abundance of Firmicutes was significantly higher and Bacteroidota significantly lower than in the control group. These results suggest that the fecal microbiota response to *L. europaeus* infection may consist primarily of a selective reorganization of taxonomic composition rather than a widespread loss of microbial diversity. Therefore, the infection did not lead to the disappearance of the basic ecological structure of the microbiota but changed the relative share of its specific components, and this effect was more visible in the GI.2 group. This difference may reflect different dynamics of *L. europaeus* infection in the two genotypes. In our model, all infected animals died, but in the GI.2 group, deaths occurred earlier than in the GI.1 group. It can therefore be assumed that the faster disruption of host homeostasis in the course of GI.2 infection could have coexisted with more pronounced changes in the composition of the microbiota. However, based on the current data, it cannot be determined whether the observed differences resulted directly from the viral genotype or were related to different infection dynamics and clinical course.

To date, data on the association of RHD with the gut microbiota of rabbits are very limited. Of particular importance is the study by Rouco et al. [18], who were the first to demonstrate an association between the fecal microbiota profile and the epidemiological dynamics of RHDV2 in natural populations of European rabbits. The authors found that the microbiota of the studied animals was dominated by Firmicutes (71 ± 15%) and Bacteroidota (19 ± 14%), and a low ratio of selected representatives of Bacteroidales to Firmicutes was associated with susceptibility to RHDV2 and was more frequently observed in animals dying during disease outbreaks. Within Firmicutes, Clostridia dominated, including Ruminococcaceae, Oscillospiraceae, and *Clostridia_UCG-014*. Our results partially correspond to these observations. In the controlled experimental model, in the GI.2 infected group, the relative abundance of Firmicutes was significantly higher than in the control group (72.7% vs. 64.0%; *p* = 0.042), whereas the relative abundance of Bacteroidota was significantly lower (17.8% vs. 26.3%; *p* = 0.042). A similar direction of changes, although without statistical significance, was also observed in the GI.1 group. These results therefore indicate a shift in the proportion of the two dominant bacterial phyla towards an increased proportion of Firmicutes relative to Bacteroidota during *L. europaeus* GI.2 infection. Furthermore, both in the study by Rouco et al. [18] and in our model, *Clostridia_UCG-014* constituted a significant component of the basal microbiota structure, although our data do not indicate a clear association of this taxon with the response to infection.

A concordant direction of changes observed in our study and in the study by Rouco et al. [18] supports the hypothesis that shifts in the relative proportions of Bacteroidota and Firmicutes may represent one component of the gut ecosystem response accompanying RHDV2 infection. However, these findings should be compared with caution. The study by Rouco et al. [18] was conducted under field conditions, where microbiota composition may have been simultaneously influenced by factors such as diet, season, age, environmental conditions, previous exposure to the virus, animal condition, and host genetic variability. Consequently, it was not possible to determine unequivocally whether the observed microbiota profile preceded infection and may have been associated with host susceptibility or whether it represented a consequence of infection or of changes in population structure resulting from mortality during the RHDV2 outbreak. The controlled experimental model used in our study reduced the influence of some of these factors, thereby providing an experimental extension of observations previously obtained under natural conditions.

Among the taxa analyzed, a particularly interesting pattern was observed for Muribaculaceae, one of the dominant families in the rabbit gut microbiota. Its mean abundance decreased from 17.3% in the control group to 11.4% in the GI.1 group and 9.0% in the GI.2 group. Although these differences did not reach statistical significance, the direction of change becomes significant when compared with correlation analysis. The abundance of Muribaculaceae was positively correlated with survival time but negatively with lethargy and diarrhea. Together, these results indicate that a higher proportion of Muribaculaceae was associated with a more favorable clinical profile of infection. These associations do not prove a protective effect of this bacterial family, but they do indicate Muribaculaceae as a particularly interesting taxon for further study of microbiological indicators of RHD. The biological significance of this observation should be considered in the context of the rabbit’s baseline gut microbiota. Combes et al. [12] include Muribaculaceae among the dominant and recurrent components of the microbiota of this species and indicate the potential contribution of this group, along with other commensal bacteria, to maintaining intestinal homeostasis and colonization resistance. The lower abundance of Muribaculaceae in infected groups may therefore reflect selective disruption of specific components of the basal microbiota, despite preserved overall diversity and a significant portion of the core bacterial community. The potential importance of Muribaculaceae may also be related to their metabolic properties. Members of this family use complex dietary polysaccharides and mucosal glycans, and their metabolism may be associated with SCFA production. These metabolites participate in the regulation of intestinal barrier function, metabolism, and the host immune response. However, the functional significance of Muribaculaceae remains dependent on the host, diet, and structure of the entire microbial community [19].

Additional support for our observations is provided by studies conducted in rabbits, indicating associations between Muribaculaceae and the metabolic and immunological environment of the gut. Li et al. [20] demonstrated associations between the abundance of Muribaculaceae and SCFA and cytokine profiles, suggesting a potential link between this taxon and metabolic and immune processes within the gastrointestinal tract. Such relationships may be particularly relevant in the context of viral infections. Liu et al. [21] emphasized that the gut microbiota may modulate antiviral immunity both through interactions with innate and adaptive immune mechanisms and through microbiota-derived metabolites, including SCFAs. In this context, we hypothesize that the decrease in the relative abundance of Muribaculaceae observed in our study during *L. europaeus* infection may be accompanied by changes in the microbiota’s fermentative potential and in metabolic and immunological interactions between the microbiota and the host. This interpretation is supported by the positive correlation between Muribaculaceae abundance and survival time, together with its negative correlations with lethargy and diarrhea, indicating that a higher relative abundance of this taxon was associated with a more favorable clinical profile of infection. However, these associations do not allow a protective role to be attributed to Muribaculaceae or the directionality of these relationships to be established. In the present study, we did not assess SCFA concentrations, intestinal cytokines, or markers of intestinal barrier integrity. Therefore, it remains unclear whether the reduced relative abundance of Muribaculaceae represents a secondary consequence of infection and the associated disruption of host homeostasis or whether this bacterial family may contribute to modulating the course of RHD.

An interesting complement to the observations regarding Muribaculaceae is the contrasting pattern observed for the relative abundance of *Sporosarcina*. This taxon was not detected in the control group, whereas its mean abundance was 0.9% in the GI.1 group and increased to 9.4% in the GI.2 group. A statistical trend was observed when the GI.2 group was compared with the control group (*p* = 0.050). At the same time, *Sporosarcina* abundance was positively correlated with diarrhea and lethargy and negatively correlated with survival time. Moreover, *Sporosarcina* and Muribaculaceae were negatively correlated with each other. Thus, Muribaculaceae and *Sporosarcina* exhibited opposing patterns of abundance and associations with clinical parameters. A higher abundance of Muribaculaceae was associated with survival time and a lower severity of selected clinical signs, whereas *Sporosarcina* reached its highest relative abundance in the GI.2 group and was positively associated with clinical signs of disease. These findings may indicate that infection progression, particularly during GI.2 infection, is accompanied by selective shifts among specific components of the microbiota. However, the present findings do not allow us to conclude that Muribaculaceae exert a protective effect or that the increase in *Sporosarcina* contributes to a more severe course of RHD. The observed associations are correlational and may represent secondary consequences of the physiological and metabolic changes occurring during infection.

The mechanisms underlying the selective changes in the microbiota during RHD remain unknown. Given that the liver is the primary target organ of *L. europaeus*, the observed microbiota alterations may also be considered in the context of the systemic consequences of infection and the associated disruption of host homeostasis. Infection leads to extensive hepatocellular damage, metabolic and coagulation disturbances, and, in severe cases, multiorgan failure [5,9,10]. One possible explanation for the observed microbiota changes is therefore an indirect effect of infection mediated by disruption of host homeostasis, inflammatory responses, and metabolic alterations, which may modify the intestinal environment and selectively affect specific bacterial groups. In this context, the gut–liver axis may also be relevant, as it represents a bidirectional communication pathway between the gut microbiota and the liver. Microbiota-derived metabolites reach the liver through the portal circulation, whereas the liver can, in turn, influence the intestinal environment through mechanisms including bile acid metabolism and immune regulation. Extensive liver damage during RHD may therefore potentially disrupt this bidirectional communication, although the relevance of this mechanism in *L. europaeus* infection requires direct confirmation. Another factor that may influence the microbiota is reduced food intake. Infected rabbits exhibited loss of appetite, lethargy, and diarrhea. In rabbits, whose gastrointestinal physiology is closely linked to hindgut fermentation and cecotrophy, even a short-term reduction in food intake may alter the availability of fermentable substrates to the microbiota. Reduced availability of fermentable polysaccharides may therefore affect bacteria involved in their metabolism and, consequently, may also alter the profile of SCFAs produced. Associations between rabbit microbiota composition and SCFA profiles have previously been demonstrated by Li et al. [20]. However, the present data do not allow us to establish the direction or causal nature of these relationships. A cautious interpretation suggests that the observed changes in the fecal microbiota may reflect a complex relationship between infection, the host response, and alterations in the gastrointestinal environment. Although the microbiota may contribute to the maintenance of host homeostasis, the present data do not allow the direction or causal nature of these relationships to be established. Further studies are required to clarify the direction and biological significance of these relationships.

To our knowledge, this study provides the first experimental data directly comparing the effects of *L. europaeus* GI.1 and GI.2 infection on the fecal microbiota of rabbits and provides an experimental extension of previous field observations. This experimental model allowed us to limit the influence of environmental factors and assess relationships among the microbiota profile, viral genotype, and the clinical course of infection. A limitation of this study is that microbiota analysis was restricted to fecal samples. Although fecal samples provide valuable information on the microbiota of the distal gastrointestinal tract, they may not fully reflect its spatial heterogeneity, particularly the microbiota of the cecum, which in rabbits is the main site of microbial fermentation. This limitation may be particularly relevant in the case of a rapidly progressive disease such as RHD. Therefore, further analyses of microbiota from different parts of the gastrointestinal tract, and in particular from the cecum and colon, are necessary to provide a more comprehensive understanding of the observed microbiota changes and to further elucidate the virus–host microbiota during RHD.

## 4. Materials and Methods

### 4.1. Animals and Housing Conditions

The study was conducted on 18 sixteen-month-old New Zealand White European rabbits (*Oryctolagus cuniculus*), Crl:KBL (NZW)/052, with a body weight of 4.0–4.5 kg and an equal sex distribution (50:50), obtained from a certified breeding facility (AnimaLab Sp. z o.o., Poznań, Poland). Upon arrival at the experimental facility, the animals underwent a three-week acclimatization period before the start of the experiment. Rabbits were housed under controlled environmental conditions in accordance with the applicable European Union regulations on the protection of animals used for scientific purposes [22] and the ARRIVE 2.0 guidelines [23,24]. Room temperature was maintained at 22 ± 1 °C, with a relative humidity of 50–60%. An independent ventilation system provided 15–20 air changes per hour. A controlled 12 h light/12 h dark cycle was maintained, with red lighting used to enable observation of the animals during the dark phase. Hay balls and wooden blocks were provided as environmental enrichment. All animals had *ad libitum* access to water and feed. Throughout both the acclimatization and experimental periods, all rabbits received the same standardized diet consisting of a complete pelleted rabbit feed (ZL-K1, ZooLab, Sędziszów, Poland) supplemented with hay. The pelleted feed contained cereal grains and cereal-derived products (wheat and maize), sunflower and soybean meals, dried alfalfa, soybean oil, sugar beet molasses, dried fruit, and a mineral–vitamin premix. The composition of the diet and feeding regimen remained unchanged throughout the study, thereby minimizing the potential influence of dietary variation on fecal microbiota composition. The experiment was conducted at the animal facility of the Pomeranian Medical University (PUM) in Szczecin, Poland. The clinical condition of both infected and healthy control rabbits was monitored throughout the experimental period. Clinical monitoring included assessment of changes in activity and behavior, appetite, body temperature, gastrointestinal and respiratory disturbances, and neurological signs. The time of onset of clinical signs, disease progression, and mortality were also recorded.

### 4.2. Experimental Infection and Study Design

After the acclimatization period, the animals were assigned to three experimental groups (*n* = 6 in each group): an uninfected control group (K marking), a group infected with *L. europaeus* GI.1 (RHDV; R marking), and a group infected with *L. europaeus* GI.2 (RHDV2; R2 marking). Animals were randomly assigned to each group. The experimental animals were infected by intramuscular injection of 1 mL of the selected virus—the *L. europaeus* RHDV/GI.1 genotype (BBI strain, Poland 2017; MG602005) for group 1 and the *L. europaeus* RHDV2/GI.2 genotype (PIN strain, Poland 2018; MN853660) [25] for group 2—while the control group 3 was injected with a form of PBS (phosphate-buffered saline) as a placebo. The beginning of the experiment was defined as the administration of antigen to rabbits and PBS to the control group at 0 h. The viruses used in the experiment were prepared at the National Reference Laboratory for Rabbit Hemorrhagic Disease (RHD) and the Department of Foot and Mouth Disease, the National Veterinary Research Institute—State Research Institute in Zduńska Wola, Poland.

### 4.3. Fecal Sample Collection

Fecal samples were collected from rabbits infected with *L. europaeus* GI.1 (RHDV) and GI.2 (RHDV2) after death (Table 1). In the GI.1 (RHDV)-infected group, samples were collected between 32 and 60 hpi, whereas in the GI.2 (RHDV2)-infected group, samples were collected between 24 and 32 hpi. Fecal samples from healthy control rabbits were collected immediately after euthanasia (Table 1). All samples were collected in sterile containers, immediately frozen in liquid nitrogen, and then stored at −80 °C until further analysis. The same sample handling and storage procedures were applied to all experimental groups.

### 4.4. DNA Extraction and 16S rRNA Gene Amplicon Sequencing

#### 4.4.1. DNA Extraction from Fecal Samples

The total genomic DNA was extracted from the stool samples using the GeneMatrix Stool NA purification Kit (Eurx Ltd., Gdansk, Poland), following the manufacturer’s protocol. The quality and concentration of the extracted DNA were assessed using a NanoDrop 2000 spectrophotometer (Thermo Fisher Scientific Inc., Waltham, MA, USA). DNA integrity was verified through 1% agarose gel electrophoresis to ensure suitability for downstream applications.

#### 4.4.2. 16S rRNA Gene Amplification and Library Preparation

V3–V4 hypervariable regions of the bacterial 16S rRNA gene were amplified using isolated DNA from individual rabbit feces through PCR. The V3–V4 region was amplified using the 341F-805R primers. PCR products were purified using a QIAquick Gel Extraction Kit (QIAGEN, Germantown, MD, USA) and analyzed using an Agilent Bioanalyzer 2100 system (Santa Clara, CA, USA). Sequencing libraries were constructed using TruSeq Nano DNA Library Prep Kits (Illumina, San Diego, CA, USA). Sequences were obtained on a HiSeq 2500 system (Illumina, San Diego, CA, USA) in a 2 × 300 bp paired-end run.

### 4.5. Analysis of Experimental Data (Microbiota Data) and Statistical Analysis

The raw paired-end reads were primer-trimmed using QIIME 2 software (version 2017.4) for data quality control. USEARCH software (version 10.0) and FLASH software (version 1.2.11) were used for clustering paired-end reads into operational taxonomic units (OTUs) based on 97% sequence identity. A Venn diagram was used to represent the similarities and differences between the three groups. Alpha-diversity analysis (richness and evenness) and beta-diversity analysis (principal coordinate analysis and non-metric multidimensional scaling) were conducted using QIIME 2 software (version 2017.4). Spearman correlation analysis was executed using the R package corrplot (version 0.84). The differences among the treatment groups were determined through the Kruskal–Wallis test and Dunn’s pairwise test using SAS software (version 9.4, 2012; SAS Institute, Cary, NC, USA). The *p* value of principal coordinate analysis (PCoA) and non-metric multidimensional scaling (NMDS) was calculated using the Bray–Curtis index. A *p* value less than 0.05 was considered significant, and a *p* value less than 0.1 was considered a trend.

## 5. Conclusions

This study demonstrated that experimental infection of rabbits with *L. europaeus* GI.1 (RHDV) and GI.2 (RHDV2), despite differences in clinical disease dynamics, was not associated with a global restructuring of the fecal microbiota but rather with selective changes in its taxonomic composition. The observed changes were more pronounced in the *L. europaeus* GI.2-infected group, which also exhibited a more rapid clinical course. Associations between the relative abundance of selected bacterial taxa, particularly Muribaculaceae and *Sporosarcina*, and clinical parameters suggest that specific components of the fecal microbiota may be associated with the course of RHD. However, given the correlative nature of these associations, it remains unclear whether the observed microbiota changes are a consequence of infection and disease progression, are part of the host response, or may themselves contribute to modulating the course of RHD. To our knowledge, this is the first controlled experimental study directly comparing the effects of *L. europaeus* GI.1 and GI.2 infection on the fecal microbiota of rabbits. Our findings experimentally extend previous field observations and offer new insights into the association between *L. europaeus* infection and the host microbiota during RHD. Muribaculaceae and *Sporosarcina* emerge as particularly interesting taxa for further investigation into their potential role as microbiological indicators of infection progression. Further studies analyzing microbiota from different parts of the gastrointestinal tract are necessary to determine the biological significance of the observed changes and to elucidate the mechanisms underlying the interactions between *L. europaeus* and the host microbiota.

## Figures and Tables

**Table 1:** Characteristics of the experimental groups and clinical outcomes following *L. europaeus* GI.1 (RHDV) and GI.2 (RHDV2) infection.

Experimental Group	Survival Time	Observed Clinical Signs	Clinical Form of RHD
Control, healthy rabbits (K) (*n* = 6)	Euthanized at the end of the experimental period	No abnormal clinical signs observed	Not applicable
*L. europaeus* GI.1 (RHDV, R) infected rabbits (*n* = 6)	32–60 hpi	Loss of appetite, diarrhea, lethargy, tachypnea, elevated body temperature, and neurological signs.	Predominantly acute
*L. europaeus* GI.2 (RHDV2, R2) infected rabbits (*n* = 6)	24–32 hpi	Diarrhea, lethargy, tachypnea, elevated body temperature, and neurological signs	Predominantly acute

hpi, hours post-inoculation; RHD, rabbit haemorrhagic disease; RHDV, rabbit haemorrhagic disease virus; RHDV2, rabbit haemorrhagic disease virus 2.

**Table 2:** Effect of *L. europaeus* GI.1 (RHDV) and GI.2 (RHDV2) infection on alpha-diversity of microbiota in the fecal contents of rabbits.

					*p* Value		
	K ^1^	R	R2	SEM	K vs. R	R vs. R2	R2 vs. K
Chao 1	62.5	67.8	65.5	0.952	0.146	0.579	0.052
Fisher	6.7	7.1	6.9	0.089	0.183	0.401	0.600
Shannon	3.0	3.2	3.1	0.032	0.133	0.315	0.499
Simpson	0.9	0.9	0.9	0.004	0.245	0.401	0.797

^1^ K = healthy rabbits; R = rabbits infected with *L. europaeus* RHDV; R2 = rabbits infected with *L. europaeus* RHDV2. Mean value representing results from 6 rabbits per treatment (*n* = 6).

**Table 3:** Effect of *L. europaeus* GI.1 (RHDV) and GI.2 (RHDV2) infection on taxonomic assignment and ranking of fecal microbiota in rabbits.

					*p* Value		
	K	R	R2	SEM	K vs. R	R vs. R2	R2 vs. K
Phylum							
Firmicutes	64.0	71.4	72.7	1.528	0.183	0.944	0.042
Bacteroidota	26.3	20.2	17.8	1.400	0.320	0.597	0.042
Cyanobacteria	4.6	2.8	3.8	0.611	0.501	0.944	0.880
Verrucomicrobiota	2.9	2.4	2.6	0.358	0.881	1	0.986
Proteobacteria	1.1	1.1	1.8	0.176	0.320	0.944	0.880
Selected bacterial taxa							
*Clostridia_UCG-014*	14.2	13.6	11.7	1.317	1	0.986	0.797
Muribaculaceae	17.3	11.4	9.0	1.424	0.245	0.063	0.795
*Ruminococcus*	7.4	7.6	6.6	0.538	0.986	0.879	0.880
Clostridia_vadinBB60_group	7.4	7.1	6.3	0.529	0.986	0.986	0.880
Lachnospiraceae	4.1	5.4	4.7	0.638	0.945	0.597	0.600
Eubacteriaceae	3.7	3.9	5.1	0.526	1	0.496	0.499
*Sporosarcina*	0	0.9	9.4	1.918	0.141	0.472	0.050
V9D2013_group	2.5	4.4	3.7	0.529	0.320	0.879	0.499
Gastranaerophilales	4.6	2.8	3.8	0.611	0.501	0.944	0.880
NK4A214_group	3.0	3.8	2.5	0.286	0.881	0.240	0.499
*Bacteroides*	3.1	3.0	2.9	0.297	0.945	0.986	0.989
*Akkermansia*	2.9	2.4	2.6	0.356	0.881	1	0.989
*Alistipes*	2.8	2.3	1.8	0.382	0.798	0.986	0.880
Lachnospiraceae_NK4A136_group	2.3	2.0	1.7	0.134	0.406	0.879	0.092
*UCG-010*	2.3	2.1	1.6	0.215	1	0.401	0.701

K = healthy rabbits; R = rabbits infected with *L. europaeus* GI.1 (RHDV); R2 = rabbits infected with *L. europaeus* GI.2 (RHDV2). Mean value representing results from 6 rabbits per treatment (*n* = 6); SEM = standard error of measurement.

## Data Availability

The original contributions presented in the study are included in the article; further inquiries can be directed to the corresponding author.
